# A comparative study on lexical and syntactic features of ESL versus EFL learners’ writing

**DOI:** 10.3389/fpsyg.2022.1002090

**Published:** 2022-11-01

**Authors:** Chao Zhang, Shumin Kang

**Affiliations:** College of Foreign Languages, Qufu Normal University, Qufu, Shandong, China

**Keywords:** Hong Kong ESL learners, EFL learners in mainland China, English compositions, secondary school students, lexical and syntactic features

## Abstract

This study analyzes the compositions of Hong Kong English as a second language (ESL) learners and English as a foreign language (EFL) learners in Mainland China in terms of lexical and syntactic features. A program based on the CoreNLP was developed and used to analyze written language texts, and differences in tags of parts of speech and syntactic dependencies between the two groups of texts were compared statistically to examine differences in the lexical and syntactic features of the learners’ written language. The results show significant differences in the lexical and syntactic features of learners’ writing. Specifically, in EFL learners’ writing, there is a salient group pattern of higher lexical diversity, whereas ESL compositions are more flexible in vocabulary use with higher information density, in that they use more syntactic phrases and content words. In terms of syntax, Hong Kong ESL students use more adverbials and adverbial clauses, which is advantageous in syntactic simplicity and readability over their counterparts, whereas Mainland China EFL students prefer using more specific expressions to demonstrate syntactic relations. Compared to EFL compositions, ESL compositions are more informative, coherent, and grammatical in lexical features and more readable in syntactic features, which require more attention and further improvements in terms of EFL teaching.

## Introduction

Lexical and syntactic features, e.g., word length, word frequency, lexical richness, part of speech, syntactic complexity, syntactic dependency, etc., can reveal the crucial characteristics of a written text (Nation, 2001; Novikova et al., 2019). Research have found that a higher-rated writing generally contains more complex and diverse lexical items and syntactic structures (Ortega, 2003; Lu, 2010; Bulté and Housen, 2014). Therefore, the lexical and syntactic features of learner output are considered reliable and comprehensive indicators of writing quality, which has been a focal area investigating language learning outcomes, such as for exploring the differences between learners’ written language in foreign and second language learning contexts.

Language learning context is considered to have a significant influence on the development of writing ability, which may be investigated by detecting lexical and syntactic differences learners’ written language. Much of the literature on lexical and syntactic features has paid particular attention to the two aspects. Some researchers have investigated the measurement of lexical richness and syntactic complexity to validate various indicators (Norris and Ortega, 2009; Lu, 2010), in which the correlations between syntactic complexity and writing quality have been investigated (van Hout and Vermeer, 2007; Ortega, 2015). Other studies have concentrated on the effect of task variables, cognitive processes, or contextual factors on the syntactic complexity of second language writing (Johnson et al., 2012; Chang and Wang, 2016; Zhou and Lü, 2022). Previous studies, however, have primarily been based on cross-sectional design comparing different groups of foreign or second language writing corpora or longitudinal studies analyzing the written language characteristics (e.g., lexical richness and syntactic complexity) of the same group of learners to explore emergent patterns of syntactic performance and learners’ syntactic development (Vercellotti, 2015), in which the participants were mainly from colleges (Vyatkina, 2012, 2013) and less frequently from secondary schools. The under-researched area concerns comparative studies on the written lexical and syntactic features of English as a second language (ESL) learner in a bilingual context and English as a foreign language (EFL) learner in an instructed language learning context. Therefore, the present study examines the lexical and syntactic features of the written language of ESL and EFL learners, especially parts of speech and syntactic dependencies and their influencing factors, aiming to explore the differences in written language between the two groups to provide some references and insights for language teaching practice.

## Literature review

### Differences between second language acquisition versus foreign language learning

One critical distinction that has been made in English learning contexts is that of the ESL context versus the EFL context (Shehadeh and Coombe, 2012). A second language learning context is one in which target language is widely used in the language society, whereas foreign language learning contexts are those where the target language is learned and used mainly in school settings, specifically in classrooms (Loewen, 2015). The ESL context, in contrast to the EFL context, is considered to have differences in L2 input and output. One hypothesis is that students exposed in second language learning context acquire considerably more L2 input in language societies. The quantity of L2 input (i.e., exposure frequency to the target language) has been highlighted by many theoretical frameworks and hypotheses in SLA academia. According to Ortega (2014), language learners can only successfully acquire L2 rules when they have reached a critical mass of exposure to the target language, which echoes the Critical Mass Hypothesis developed by Marchman and Bates (1994) who found that proficient learners, in lexical and morphological development, needed to reach a “critical mass” of L2 exposure. ESL learners are more likely to reach the critical mass than EFL learners, in that they can easily obtain access to a greater amount of the target language. Research findings also suggest that ESL learners may indeed be exposed to more of the target language due to language policy and pedagogical factors. L2 teaching in the ESL context focuses more on meaning and fluency than structure, tending to provide a wide range of content-related spoken language (Vold, 2022).

ESL learners may also have more opportunities to communicate with L2 speakers, which is considered to be crucial for second language learning. In contrast to EFL learners, ESL learners enjoy considerably more interactions with L2 speakers, in which the “meaning negotiation” occurs incessantly and unremittingly (Long, 1981). Ortega (2014) generalized the Language Exposure Hypothesis, claiming that the prerequisite of L2 acquisition is language exposure, emphasizing the critical function of language exposure for L2 learning. According to the Input Hypothesis, the L2 input of high quality is “comprehensible input,” which is slightly more advanced than the current level of learners’ proficiency (i + 1; Krashen, 1985). The meaning negotiation process provides “comprehensible input” for L2 learners, which undoubtedly enhances the quality of their L2 input and output (both spoken and written).

ESL and EFL should not be seen as two completely discrete systems of English categories. Buschfeld and Kautzsch (2017) developed the model of Extra-and Intra-territorial Forces (EIF model), which is an integrative language model that EFL and ESL are situated on a continuum with interconnectedness in ethnicity, society, proficiency level, age, etc. ESL and EFL form a complex and integrated system. Nonetheless, the EIF model is compatible with other second language acquisition theories (e.g., Critical Mass Hypothesis and Input Hypothesis), emphasizing the crucial influence of language learning context on language development. However, according to the EIF model, differences of writing performance arising from language acquisition contexts should be analyzed from a more integrated perspective, that is, to consider a wider range of influencing variables (e.g., political, social, and educational factors) and their interactions (Buschfeld, 2013; Buschfeld and Kautzsch, 2020).

From a theoretical perspective, learning context has a significant impact on L2 acquisition. Whether there are differences in written features of output between ESL learners in a target language context and EFL learners in non-target language context is a matter of inquiry in empirical research.

### Empirical research on the effect of learning context on L2 writing

Empirical research on the effect of learning context on L2 writing has rested primarily on the assumption that the learning context (e.g., ESL or EFL) impacts the acquisition process and thus the quality of second language writing (Loewen, 2015). Several lines of empirical evidence have suggested that learners’ L2 lexical and syntactic development is related to exposure mode and frequency use of language (Vercellotti, 2015; Nasseri and Thompson, 2021; Huang et al., 2022). However, others have found contradictory results on the effect of learning context (Bardovi-Harlig and Bergstrom, 1996; Lyster and Saito, 2010). Few comparative studies have concentrated on lexical and syntactic features of second language versus foreign language learners’ writing. Håkansson and Norrby (2010), for example, examined written language features of college Swedish learners, and found written differences between second and foreign language learners in pragmatics and lexicon. However, empirical research findings from adjacent research areas (e.g., spoken output research) have provided mixed evidence on the effect of learning context on L2 proficiency (Bardovi-Harlig et al., 1998; Kasper and Rose, 2002; Schauer, 2006; Charkova and Halliday, 2011; Vold, 2022). Schauer (2006) found that the college EFL learners were less aware of pragmatic items than the ESL learners. Similarly, Charkova and Halliday (2011) investigated the effect of ESL and EFL contexts on tense backshifting in indirect reported speech, finding that EFL context facilitates college learners automated the use of tense backshifting, whereas the ESL context fostered awareness of tense backshifting. However, Bardovi-Harlig and Bergstrom (1996) conducted a cross-sectional study and found that learning context may influence the acquisition in terms of tense or aspect in L2 writing. Evidence from meta-analyses also suggests that the influence of the learning context on language acquisition is controversial. Researchers have identified that the mediating effect of learning context is significant for the effectiveness of corrective feedback and interaction (Mackey and Goo, 2007; Li, 2010), whereas other meta-analyzed studies also revealed the insignificant mediating effect of learning context on the L2 acquisition effect (Lyster and Saito, 2010). These findings offer mixed results on the effect of learning context on L2 learning and an under-researched area in terms of the lexical and syntactic features of ESL and/versus EFL learners’ writing, which may arise from the complexity of lexical and syntactic features interconnected with subsystems of language development. Therefore, exploring syntactic and lexical features contributes to a more integrated understanding of the development of these aspects of written text.

Previous studies into the development of learners’ written language have primarily used holistic indicators with a single dimension, i.e., by analyzing a specific lexical and syntactic feature (e.g., word length, sentence length, lexical density, phrase complexity, or the proportion of parallel and subordinate structures) to examine learners’ lexical and syntactic features and trajectories of L2 development (Ortega, 2003; Lu, 2010; Youn, 2014; Atak and Saricaoglu, 2021; Maamuujav et al., 2021). Research findings show that different-level learners prefer using different lexical and syntactic structures in their written work, with beginners using high-frequency vocabulary, parallel structures, and simple sentences, intermediate learners tending to use low-frequency vocabulary and subordinate clauses, and advanced learners often using simple language structures (e.g., noun phrases) to express more complex ideas (Ortega, 2003; Vyatkina, 2013). Similarly, recent studies have found that there are significant differences in specific syntactic indicators between L2 learners and native speakers, with bilingual learners tending to use more parallel structures and native speakers tending to use more subordinate and complex phrase structures (Bulté and Housen, 2014; Mancilla et al., 2017; Shadrova et al., 2021). Notwithstanding difference in learners’ level of syntactic development, high-quality compositions shared similar linguistic features, such as the use of more complex sentences and diverse phrase structures, as well as low-frequency vocabulary to convey meaning (McNamara et al., 2010). Few studies have been conducted on learning context focused on learners’ lexical and syntactic features. Håkansson and Norrby (2010) investigated the difference in terms of lexical and grammatical features between the groups based on learning context, also focusing on the development of several lexical and syntactical features, e.g., associations, noun phrase agreement, subject-verb inversion, and predicative agreement, which may not further explore language development differences across both groups of learners in terms of more critical features (e.g., parts of speech and syntactic relations). Which specific lexical forms and syntactic structures do different learners tend to use? These areas have not been properly explored in existing research.

In sum, three under-researched areas remain. Firstly, there is a learning context hypothesis that requires verification. However, previous empirical studies have primarily been conducted in the homogenous learning context, with few studies comparing the written syntactic features of ESL and EFL learners, yielding an insufficient understanding of the effect of language learning context. The distinction between ESL and EFL is particularly critical as it affects whether the findings in one context can be generalized to another (Loewen, 2015; Loewen and Sato, 2017). Secondly, existing studies have primarily used indicators of lexical richness and syntactic complexity, focusing on lexical diversity, sentence length, word frequency, and a few syntactic features such as noun agreement and subject-predicate inversion, and paying insufficient attention to other more critical lexical and syntactic features. Using a small number of indicators leads to a bias where only the indicators used in the study can be observed, and more extensive differences cannot be examined. This focus leaves an under-explored area, namely, which parts of speech and syntactic relations are responsible for these differences in written language? Thirdly, previous studies have focused primarily on college student populations, with insufficient attention being paid to the cohort of secondary school students. Thus, as an attempt to address such challenge, the present study incorporates lexical and syntactic features as a measurement dimension, aiming to acquire a more comprehensive understanding of the difference in the lexical and syntactic features of ESL and EFL learners’ textual output.

## Methodology

This study used a comparative design to investigate the differences in written language in Hong Kong ESL and mainland China EFL contexts, aiming to explore their differences and influencing factors by comparing the English composition texts of high school students in the two regions and analyzing the statistical characteristics of the two groups of learners at a lexical and syntactic level.

### Research questions

The following research questions have been formulated:

(1) What are the lexical differences between the two groups of participants’ writing?

(2) What are the syntactic differences between the two groups of participants’ writing?

### Participants and their learning contexts

The participants were 62 senior secondary school students from two secondary schools in Hong Kong and Jiangsu (southern province in eastern China). All participants were high school seniors and were of similar age (17–18 years old), who had been studying English in the instructed context for the same number of years. Thirty-two participants in Hong Kong were ESL learners (Chan, 2011; Luk and Hui, 2017) and 30 participants in Jiangsu were EFL learners. In the present study, ESL refers to English as one of the two official languages with a recognized social status widely used in politics, economics, education, etc., whose treatment is similar to or even surpasses that of the mother language, Chinese in Hong Kong. However, EFL refers to English as a non-official foreign language learned in Mainland China through formal classroom instruction in a native-speaking social context (Joseph, 2020).

The two groups of participants are at the same level of schooling in terms of English learning. However, the learners are exposed to different pedagogical resources, teaching contexts, and instructional strategies. As an official language, English has been dominating the executive, legislative, commercial, and educational spheres of Hong Kong since 1842. After the handover, the Hong Kong SAR Government implemented a policy of “Biliteracy and Trilingualism” in language education, with “biliteracy” referring to written Chinese and English, both of which are official languages, and “trilingualism” referring to Mandarin, Cantonese, and English. In this context, documents issued to the public (e.g., government work reports, official documents, curriculum documents, etc.) are available in English. In the education sector, Hong Kong continues the educational convention and language usage habits of the British Hong Kong period, with schools using English textbooks for the English Language curriculum (known as “British Language” in Hong Kong). Furthermore, textbooks for other subjects (e.g., Mathematics, Science, Physics, Chemistry, Computer and Information Technology, etc.) are used in English or bilingual, depending on the school. Therefore, the social context and use of English as an official language provide ESL learners in Hong Kong with more opportunities to access and use English than EFL learners in Mainland China. Moreover, ESL learners’ language input and output patterns in their learning context approximate the natural acquisition mechanism or learning process of native English speakers (Spolsky, 1989; Ortega and Han, 2017). Therefore, how the lexical and syntactic features of written English differ among high school students from the two regions in different learning and pedagogical contexts is an under-researched area.

### Indicators of lexical and syntactic features

In order to compare differences between the two groups in terms of the lexical and syntactic features of their written English, this study analyzes written English features in terms of aspects of lexis and syntax. The lexical analyses involve the part of speech (the ratio of part of speech to words), which represents the lower-level lexical rules. The syntactic dependencies reflect the relations above the phrasal level (e.g., clauses; Rauh, 2010). In this study, the Penn Treebank was used for the measurement of syntactic dependency analysis (Nivre et al., 2016), covering all of the indicators required for syntactic structure analyses, which has high validity in English syntactic analysis research (Lu, 2010). Lexical diversity is measured using type-token ratio (TTR), which is a valid lexical indicator. However, TTR is affected by the length of the text. In order to eliminate the deficiency of TTR, the Uber index was used in this research to measure lexical diversity, which is a variant of the TTR calculated as Uber = (log Tokens)^2^/(log Tokens-log Types; Jarvis, 2016).

### Data collection

Data were collected from essays written by participants of the same cohort in Hong Kong and Mainland China (Hong Kong S6 and Senior Secondary in Mainland China) under the same conditions. The task of the essay was a letter of invitation to introduce a cultural exchange program in the form of a timed practical writing with no less than 200 words. The writing was based on brief textual prompts, without any interference of external “evaluation,” to ensure that participants carried out the writing task in an authentic context.

### Data analysis

**Figure 1:**
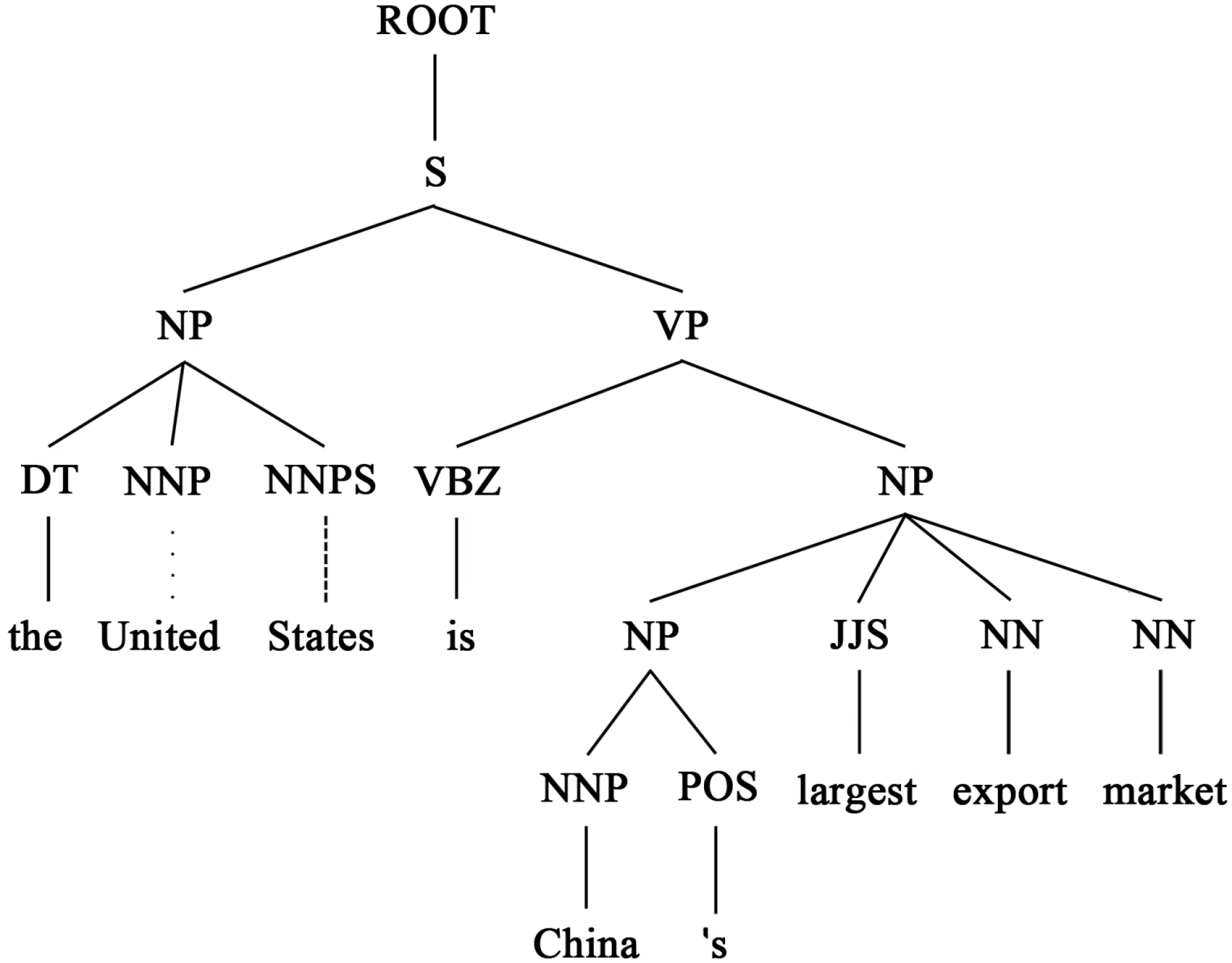
A sample of syntactic constituents (part of speech) analysis.

The analysis was conducted as follows. Firstly, participants’ written work was manually transcribed into TXT files and double-checked for accuracy. The transcribed text was then analyzed using CoreNLP (Manning et al., 2014) developed by the Stanford team, which can output strings of syntactic constituent (hierarchical lists of parts of speech) and lists of syntactic dependencies (syntactic features). The syntactic analysis results were then manually checked sentence by sentence to correct errors. For example, the syntactic constituent analysis of the sentence “The United States is China’s largest export market output” by CoreNLP is shown in Figure 1.

The first level of syntactic structure is the phrasal level, below which is the lexical level of parts of speech. In the sample syntactic structure analysis in Figure 1, the sublevels of the noun phrase (NP) are the determiner (DT) and *United States* (proper noun phrase, NNPS), whereas the sublevel of the verb phrase (VP) consists of *is* (third person of the verb in the present tense, VBZ) and the noun phrase (*China’s largest export market*). The syntactic constituent analysis of Figure 1 is parsed into the following list of syntactic dependencies.

[(‘ROOT’, 0, 9), (‘det’, 3, 1), (‘compound’, 3, 2), (‘nsubj’, 9, 3), (‘cop’, 9, 4), (‘nmod: poss’, 9, 5), (‘case’, 5, 6), (‘amod’, 9, 7), (‘compound’, 9, 8), (‘punct’, 9, 10)].

Each tuple in the list represents a set of syntactic dependencies. For example, *the* (1) and *States* (3) form a syntactic relation of determiner modifier (det), whereas *United* (2) and *States* (3) form a compound relation (compound). Dependency relations reveal links between sentence components. In the present study, the differences in the number of syntactic structures (e.g., VP, NP, and NNP) and syntactic dependency markers (e.g., det, compound, etc.) between the two groups of texts were counted in statistics, and the differences in the number of these markers offer an overview of syntactic use preferences of the learners in the two regions. A Python program was developed to count the number of lexical and syntactic indicators per-unit word count in the text, after which an independent sample *t*-test was conducted using SPSS to assess the statistical significance of the syntactic differences between the two groups’ written language.

## Results

To determine whether there were lexical and syntactic differences between the written language of Hong Kong ESL and Mainland EFL learners, as well as specific manifestations of these differences, lexical and syntactic measures according to the Pennsylvania Treebank were counted. There were no significant differences in some syntactic and lexical items (e.g., quantifier, possessive, direct object, coordination, relative clause modifier, etc.), though there were significant differences in some items. The following section highlights the main findings on the differences between the written languages of the two groups.

### Lexical features

At a lexical level, the results of the part of speech analysis show that the written English of Hong Kong ESL and Mainland EFL learners differed significantly across several dimensions (Table 1). Specifically, Hong Kong ESL learners maintained an advantage in several aspects, with a higher proportion of nouns (*M* = 0.248; SD = 0.032), connectives (*M* = 0.025; SD = 0.012), determiners (*M* = 0.071; SD = 0.022), prepositions and subordinating conjunctions (*M* = 0.096; SD = 0.018), adjectives and ordinals (*M* = 0.078; SD = 0.022), adverbs (*M* = 0.053; SD = 0.022), adverbial comparatives (*M* = 0.002; SD = 0.003), to-markers (*M* = 0.026; SD = 0.014), and wh-adverbs (*M* = 0.005; SD = 0.006) in their written English. Mainland EFL learners, however, used more diverse words with higher Uber index (*M* = 22.115; SD = 3.271).

**Table 1:** Lexical differences in written English between learners of the two regions.

Constituent indicator (code)	Jiangsu (*N* = 30)		Hong Kong (*N* = 32)		*t*
	*M*	SD	*M*	SD	
Noun	0.173	0.094	0.248	0.032	−4.169**
Connective	0.015	0.013	0.025	0.012	−2.946^**^
Determiner	0.051	0.031	0.071	0.022	−2.986^**^
Preposition and subordinating conjunction	0.043	0.030	0.096	0.018	−8.562^**^
Adjective and ordinal	0.052	0.033	0.078	0.022	−3.606^**^
Adverb	0.028	0.017	0.053	0.022	−5.174^**^
Adverbial comparative	0.000	0.002	0.002	0.003	−2.678^**^
To- markers	0.013	0.013	0.026	0.014	−3.725^**^
Wh-adverbs	0.000	0.001	0.005	0.006	−4.992^**^
Uber index	22.115	3.271	18.118	3.073	4.985^**^

** *p <* 0.01.

Learners from the two regions showed written differences in nouns (*t* = −4.169), connectives (*t* = −2.946), determiners (*t* = −2.986), prepositions and subordinating conjunctions (*t* = −8.562), adjectives and ordinals (*t* = −3.606), adverbs (*t* = −5.174), adverbial comparatives (*t* = −2.678), to-markers (*t* = −3.725), wh-adverbs (*t* = −4.992), and Uber index (*t* = 4.985). Furthermore, lexical indicators differed in ratio to a statistically significant level (*p* < 0.01). The lexical features characterize the underlying structure of sentences and demonstrate differences in part of speech preference and depth of vocabulary knowledge in written English between learners from different language learning contexts.

### Syntactic features

The results of the dependency analysis suggest that the written English of Hong Kong ESL and Mainland EFL learners differed significantly in six syntactic structure aspects (Table 2). Specifically, Mainland China EFL learners used more auxiliaries (*M* = 0.050; SD = 0.015), clausal complements (*M* = 0.017; SD = 0.010), parataxis (*M* = 0.017; SD = 0.017), and indirect object structures (*M* = 0.003; SD = 0.004), whereas Hong Kong ESL learners used fewer auxiliaries (*M* = 0.038; SD = 0.013), clausal complement structures (*M* = 0.011; SD = 0.009), parataxis (*M* = 0.008; SD = 0.009), and indirect object structures (*M* = 0.001; SD = 0.003) in their written English. The difference in the use of auxiliaries between the two groups of learners was highly significant (t = 3.576; p < 0.01), as well as in the use of clausal complements (*t* = 2.591, *p* < 0.05), parataxis (*t* = 2.672, *p* < 0.05), and the indirect object (*t* = 2.355, *p* < 0.05).

**Table 2:** Syntactic differences in written English between learners from the two regions.

Dependency indicator	Jiangsu (*N* = 30)		Hong Kong (*N* = 32)		*t*
	*M*	SD	*M*	SD	
Auxiliary	0.050	0.015	0.038	0.013	3.576^**^
Adverbial modifier	0.061	0.017	0.061	0.023	−2.245^*^
Adverbial clause modifier	0.006	0.006	0.013	0.008	−3.980^**^
Clausal complement	0.017	0.010	0.011	0.009	2.591^*^
Parataxis	0.017	0.017	0.008	0.009	2.672^*^
Indirect object	0.003	0.004	0.001	0.003	2.355^*^

* *p* < 0.05;

** *p* < 0.01.

In contrast, Hong Kong ESL learners used more adverbial modifiers (*M* = 0.061; SD = 0.023) and adverbial clause modifiers (*M* = 0.013; SD = 0.008) in their written English, whereas Mainland EFL learners used fewer adverbial modifiers (*M* = 0.061; SD = 0.023) and adverbial clause modifiers (*M* = 0.013; SD = 0.008). The difference in the use of adverbial modifier (*t* = −2.245; *p* < 0.01) and adverbial clause modifier (*t* = −3.980; *p* < 0.05) between the two groups was highly significant.

## Discussion

This study analyzed the lexical and syntactic features of the compositions of Hong Kong ESL and Mainland Chinese EFL learners. There were significant differences between the two groups of learners in terms of lexical and syntactic features, with the emergence of group patterns in different learning contexts.

### Lexical features of ESL learners: more informative, more coherent, and more grammatical

Group characteristics did not emerge among Mainland EFL students’ written English in the lexical dimension. On the other hand, Hong Kong ESL students’ written English showed significant features in terms of specific parts of speech. Part of speech reflects vocabulary use preference at the lexical level. There were more nouns, verbs, adjectives, adverbs, and prepositional phrases in Hong Kong students’ writing, indicating a richer syntactic structure in their written English. Specifically, Hong Kong students’ written English showed four significant features.

Firstly, Hong Kong ESL students use more nouns, adjectives, adverbs, and comparative forms of adjectives and adverbs, as well as ordinal. Nouns, adjectives, and adverbs are content words, and texts with a higher proportion of content words (i.e., higher lexical density) carry greater linguistic information, reflecting the quality of the information conveyed in the composition (Biber and Gray, 2016). As the most basic constituent of language, vocabulary, particularly content words, and phrase collocations, contributes to the achievement of the communicative purpose of language output and the acceptable expression of the semantic system. Hong Kong students’ compositions have more syntactic phrases (noun phrases, verb phrases, adjective phrases, adverb phrases, and prepositional phrases) and content words and determiners, resulting in more information-dense, concise syntax, making their language output more grammatical and highly informative (Rossi et al., 2006; Tolentino and Tokowicz, 2011).

Secondly, these learners’ texts contain more connectives. Connectives are an indicator of writing fluency in achieving syntactic and semantic logical coherence. The bonding effect of connectives enhances the cohesion and coherence of discourse, indicating that Hong Kong ESL students’ writing fluency is higher in conveying English morphological information. Due to the use of connectives, Hong Kong students’ written English is closer to the features of native English writers in morphology. This text is more readable with less cognitive load and requires less effort for readers to understand the meaning (Crossley et al., 2008; Chang and Wang, 2016).

Thirdly, a larger number of determiners were used in Hong Kong EFL students’ written English. Determiners, in conjunction with nouns, qualify the meaning of a noun in terms of morphological, generic, or quantitative aspects, which have specific and qualifying grammatical functions (Heusinger et al., 2019). Determiners are challenging to learn for Chinese learners of English in that there is a lack of distinct determiners in Chinese (Chang, 2017), particularly when there are multiple determiners in a noun phrase and when the position of determiners in a phrase (e.g., anterior, medial, or posterior) must be determined according to collocation rules. Thus, the use of determiners in accord with the context improves the accuracy of meaning expression and language output quality.

Fourthly, the text contains more prepositions, to-constructions, and wh-adverbs. Prepositions in English are used not only to mark time and place, but also are crucial components of phrases and collocations, to-construction are a preposition and an infinitive. Furthermore, wh-adverbs can be used in interrogative sentences and adverbial clauses. The acquisition of the combination of these words is challenging, and their practical use is more complex, reflecting learners’ writing competence on vocabulary and syntactic structures. Learners must acquire conceptual knowledge and grammatical rules of the target language and produce acceptable (i.e., native-like) forms of expressions in the target language, all of which derive from the comprehensive use of the conceptual knowledge of the target language (Mitchell et al., 2019).

Fifthly, Hong Kong students use less diverse vocabulary in their compositions, which shows the complexity of ESL and EFL systems, as well as the continuum feature of those systems. It also demonstrates the necessity and advantages of using multivariate index analysis, as designed in this study. Furthermore, the higher lexical diversity of EFL learners’ writing might relate to the top-down constrained learning and teaching context. Literature analysis reveals that the strengths of Mainland EFL students in lexical diversity might directly relate to the learning requirements of the curriculum and the way in which language knowledge is taught and learned. The objectives of English vocabulary knowledge in the Mainland are clear. The New Course Syllable for English issued by the China Ministry of Education stipulates the vocabulary mastery at each level of senior secondary school. This educational orientation prompts an extensive training and exogenous methods of vocabulary learning in English language teaching, which has led learners to use a greater variety of vocabulary in their compositions. In contrast, the eight Key Learning Area Curriculum Guides (from Primary 1 to Secondary 6) recommended by the Curriculum Development Council of Hong Kong present general language knowledge objectives, in which each unit of the textbooks lists about 15 commonly used words, without specific requirements for the mastery of vocabulary. This educational orientation has led to the use of more implicit and incidental approaches to vocabulary acquisition in English language teaching.

### Syntactic differences between ESL and EFL learners’ written language: readable versus structured

In terms of syntactic features, Hong Kong ESL students’ written language contained more adverbial modifiers and clause modifiers. However, mainland EFL students preferred to use more auxiliary, subordinate clause complementary, parataxis, and indirect object syntactic structures in written English. Written text with more auxiliary verbs, subjunctive complements, and indirect object relations made discourse more structured seemingly but reduced L2 simplicity and flexibility to a certain extent. Excerpt, Excerpts 3, and Excerpt 5 are from Mainland students’ English essays and Excerpt 2 and Excerpt 4 are from Hong Kong students’ English essays. Excerpt 1 and Excerpt 3 show the tendency of Mainland EFL students to retain indirect objects when introducing a cultural exchange project. In contrast, in Excerpt 2, the Hong Kong ESL student has omitted the indirect object for a more concise expression as the indirect object in the context of letters is generally definite. Furthermore, the Mainland EFL students’ written English shows the feature of syntactic dominance (Excerpts 1, 3, and 5), with semantics attached to syntactic dependencies, lacking extensibility and flexibility. Specifically, the use of the “so that” complementary clause, attributive clause (e.g., which is a culture exchange program), and indirect objectives reduces the simplicity of the expression. The Hong Kong ESL students prefer to use adverbial modifiers to connect the adjacent components (Excerpt 2), replacing the “so that” clause with the to-infinitives. As such, the sentences were simple and native-like, with an emphasis on conveying information and demonstrating semantic dominance.

Excerpt 1 (Jiangsu EFL learner): I want to introduce a culture exchange programme for you. Firstly, we can know more about different country’s history, culture and others knowledge. Secondly, we can get friendship and expand social round.

Excerpt 2 (Hong Kong ESL learner): I am writing to introduce a cultural exchange programme which connects schools in different countries. In this activity, students will be able to get to know each other through special projects to share information and learn more about each other’s culture.

Excerpt 3 (Jiangsu EFL learner): I’m intended to introduce the Young World which is a culture exchange programme to you. The programme has lots of strength. First, it can open our minds and our knowledge will be improved. Second, it will entertain our ability to English and contact our ability to other people.

Excerpt 4 (Hong Kong ESL learner): What do you think about fashion? The miniskirt, jersey and dungarees are beauty, is not it? If you agree that, come and enjoy our charity fashion show! This charity fashion will take place on the school hall and will be held on October at 4:00 pm. And the ticket price is $20 per students, $80 per parents and $100 per public.

Excerpt 5 (Jiangsu EFL learner): We can practice speaking English so that we can increase our English. English learning is important now. Do not need too much pressure. Join!

Mainland EFL students’ written English consists of more parataxis forms, that is, there are fewer connectives in their written English. Excerpt 5 shows a syntax is influenced by Chinese, in which there is not only a sense of non-connectives, but also inconsistency with native English, with *“increase”* and *“need”* demonstrating improper collocation. Similar improper collocation also appeared in the verb-object clauses in Excerpt 1 and Excerpt 3 (e.g., entertain/contact ability). This explicit inappropriateness, e.g., missing connectives and improper collocation, as well as implicit inappropriateness, may emerge from the learning transference of the linguistic form of English in conceptual structures of the mother language (i.e., Chinese), in that evidence from Systemic Functional Linguistic research shows a transformable relationship between parataxis and hypotaxis for Chinese writers (Li and Yu, 2021).

Language output is the selective expression of learners’ linguistic resource in an ESL or EFL context, stemming from the implicit input of the authentic language context and the way in which language rules are memorized and acquired (Ellis, 2003). The differences in syntactic structure between the two groups of learners reflect the mapping effect of the differences between ESL and EFL in terms of pedagogical environment, language input, and learning purposes. Second language acquisition is a usage-based cognitive process in which the higher frequency of language exposure draws learners’ more attention, the formation of conceptual structures for language output, and the development of linguistic forms consistent with the target language (Ellis, 2008; Tyler and Ortega, 2018). For historical reasons, English has a strong official status in Hong Kong, and the ESL context may provide Hong Kong English learners with a large amount of authentic language input, subconsciously influencing learners’ language cognition, linguistic knowledge, and way of thinking, emerging as the basis for written expressions and syntactic development (Warren, 2019). The language output or language construction process of ESL learners relies, to some extent, on naturally acquired conceptual knowledge, subconsciously using both implicit knowledge of language acquired incidentally in everyday life (idiomatic usage and sentence structure) and creative combinations of explicit knowledge learned in the classroom (linguistic and semantic rules).

Compared to ESL learners, EFL learners lack target language resources and a relatively authentic language context, as well as opportunities to use the target language. English teaching in the classroom context concentrates on grammatical rules, in which learners acquire explicit grammatical knowledge. Furthermore, the China National English Curriculum Standards for High School (2017) stipulates three levels of language knowledge objectives, with a list of grammatical items and detailed explanations on the grammatical objectives at each level. Textbooks from Mainland China list syntactic learning objectives and unit sub-objectives and systematic syntactic exercises, to ensure that students are exposed to various syntactic items, progressively complete syntactic learning, and produce grammatically accurate sentences. However, it is still difficult for EFL learners to make multi-directional connections between conceptual and syntactic structures and contexts (Swain, 1995), and produce grammatical and contextual language. As such, the language output of EFL learners reflects their linguistic competence to a larger extent than their pragmatic competence (Loewen and Sato, 2017).

Lexical and syntactic features may significantly influence other aspects of written text, e.g., text readability. Readability refers to “the ease with which a text document can be read and understood” (Tanaka-Ishii et al., 2010, p. 204), an indicator in measuring the comprehensibility of a text (Crossley et al., 2011). The linguistic indicators of text readability concern lexical (e.g., number of connectives) and syntactic features (e.g., number of subordinate clauses; McNamara et al., 2014). The degree of simplicity of different syntactic forms in a discourse may reduce the complexity of syntactic structures and the effort required for reading comprehension (Crossley et al., 2008). Syntactic simplicity is the conciseness of syntactic structures and relations within a discourse; texts with a higher indicator are considered more readable (Eslami, 2014). Similarly, the use of simple syntax (e.g., short sentences, coordinate clause, etc.) in writing can improve text readability (Hinkel, 2003), whereas the extensive use of complex sentences makes it more difficult for readers to understand the meaning, which reduces the readability of the text (Biber and Gray, 2010). Studies focusing on readability for ESL/EFL learners writing on influencing factors (Betts, 1949), indicators of readability (Graesser et al., 2011), and readability analysis of elementary education textbooks (Guven, 2014) have found a potential moderating effect of lexical and syntactic features on text readability. The written syntactic features of Hong Kong ESL students make their English more readable. The results of syntactic analyses show that text readability of Hong Kong students’ written English is higher in conciseness and coherence of their writing, primarily reflected in the two aspects. Firstly, these learners’ written English is more coherent. One of the key measures of bilingual output is the extent to which the construction of the learners’ linguistic knowledge system matches the grammatical rule of the target language (Mitchell et al., 2019; Tian et al., 2022). Secondly, these learners’ written English is simpler in syntactic structures, which makes the text more accessible. The textual analyses of differences in written English readability between the two groups found that Hong Kong learners had some advantages in syntactic conciseness over counterparts in Mainland China, that is, Hong Kong learners’ written English was less redundant.

In conclusion, in terms of learners’ written language output, readability features reflect intra-sentential fluency and inter-sentential fluency, which stems from the mixed effect of language representation and cognitive process. The lexical and syntactic features of Hong Kong ESL students’ written English are partially in line with the Language Exposure Hypothesis, which argues that sufficient exposure to target languages (including exposure to target languages in a social context), especially the accumulation of linguistic knowledge in the specific context, can facilitate the transformation of static declarative knowledge and the production of more grammatical written language. Their strength in this regard is due to the accessibility of language education resources on the one hand, and the distinctive writing teaching practices of Hong Kong on the other, i.e., writing training strategy based on discourse materials and task- and process-based writing instruction (Jones, 2009), which offer insights and implications for language teaching in general and foreign language teaching in particular.

## Conclusion

This study analyzed differences in written language between ESL and EFL learners in terms of in lexical and syntactic features. The interconnectedness of vocabulary and syntax is a dimension used to distinguish the quality of compositions or measure learners’ linguistic performance quality (Rauh, 2010; Crossley and McNamara, 2012), reflecting learners’ language competence and information processing ability (Bulté and Housen, 2014). The syntactic differences in the written language of the two groups of learners, to some extent, reflect the effect of the implicit input of the language learning context and English language education intervention in both regions. Despite it being customary to refer to ESL and EFL learning as ‘Second Language Acquisition’ in academia, there are practical differences in the accessibility of English educational resources and language exposure, as well as in the teaching strategies and learning instructions of the target language, from which group differences of syntactic features of learners’ language output emerge. Probabilistic group language characteristics reflect emergent patterns in learners’ language development and have explanatory power for language development.

The findings of the study have implications in understanding the mechanism of the role of language learning context, interlingual interaction, and teaching styles in the development of a second and foreign language, thereby enhancing understanding of the laws on language acquisition in order to target and develop language output skills of ESL and EFL learners. As such, it is recommended that EFL teaching concentrates on consciously enhancing students’ exposure to language and their ability to generalize information. Teachers could provide more authentic language materials and resources to EFL learners to accumulate their linguistic knowledge and rhetorical skills, which will allow them to perceive the diversity and authenticity of language forms and compensate for the lack of authentic language environment as much as possible. Furthermore, the design of contextualized writing tasks and strengthened extended writing training based on reading materials allow students to use linguistic knowledge to express ideas in appropriate syntax and grammatical forms based on the language learning context and content (Xie and Lv, 2022), enhancing the variety of syntactic structures and the fluency of their writing, as well as their communicative competence.

It should be pointed out that this study is a tentative experiment delimited to language forms of two groups of participants’ writing. The generalizability of these results is subject to certain limitations in terms of the learning contexts. Concerning the categorization of the ESL learning context of Hong Kong, we have adopted the view that ESL learning context is one that English is an official language with a wide use in society, which can potentially provide learners in Hong Kong with more opportunities to access and use English than learners in Mainland China (e.g., Chan, 2011; Luk and Hui, 2017; Huang and Yip, 2021). The ESL context in Hong Kong, however, is somewhat distinctive in that it differs from that of native English-speaking countries in terms of English exposure, which may concern the generalizability of the study results. More studies are needed to make further exploration from the perspective of diverse learners in different learning contexts. Specifically, future research may investigate the communicative function of language and identify implicit discourse and discourse argument structures (Kormos, 2011), as well as the contextual and pragmatic aspects of language production (Abrams and Byrd, 2016). Said research may also explore the relationship between language form and composition quality and the factors that influence ESL and EFL learners’ language production, aiming to reveal general patterns of second and foreign language output and development to provide empirical support for theoretical and practical exploration of language education.

## Data availability statement

The original contributions presented in the study are included in the article/supplementary material, further inquiries can be directed to the corresponding author.

## Author contributions

CZ organized the database, performed the statistical analysis, and wrote the paper. SK contributed to conception and design of the study. All authors contributed to the article and approved the submitted version.

## Funding

This study was supported by the National Social Science Fund of Education of China (no. BEA180110).

## Conflict of interest

The authors declare that the research was conducted in the absence of any commercial or financial relationships that could be construed as a potential conflict of interest.

## Publisher’s note

All claims expressed in this article are solely those of the authors and do not necessarily represent those of their affiliated organizations, or those of the publisher, the editors and the reviewers. Any product that may be evaluated in this article, or claim that may be made by its manufacturer, is not guaranteed or endorsed by the publisher.
